# Recent advances in CAR T-cell therapy for lymphoma in China

**DOI:** 10.1007/s12094-023-03153-1

**Published:** 2023-04-16

**Authors:** Yuxuan Che, Xiuhua Sun

**Affiliations:** Department of Oncology, The Second Hospital of Dalian Medical University, 467 Zhongshan Road, Dalian, 116021 People’s Republic of China

**Keywords:** Lymphoma, CAR T-cell therapy, Clinical trials, China

## Abstract

Lymphoma is a hematologic malignancy which mainly consists of Hodgkin lymphoma (HL) and non-Hodgkin lymphoma (NHL). Although systemic chemotherapy, radiotherapy, and other advanced therapeutics, including rituximab or immune checkpoint inhibitors, have improved the prognosis in recent decades, there are still a number of patients with relapsed or refractory (R/R) lymphoma with a poor prognosis. Chimeric antigen receptor (CAR) T-cell therapy has provided a curative option for patients with relapsed or refractory lymphoma. Numerous clinical trials have been conducted worldwide and presented inspiring results that give insight into this breakthrough therapy. The development of cancer cell therapy in China has been rapid in the past years and dominates the field with the USA. This review aims to summarize the published results of CAR T-cell therapy alone or in combination with other therapies in mainland China, both in R/R NHL and R/R HL.

## Introduction

Lymphoma is a heterogeneous disease mainly comprising Hodgkin lymphoma (HL) and non-Hodgkin lymphoma (NHL), which collectively account for approximately 4.2 per 100,000 in incidence and 2.2 per 100,000 in mortality in mainland China [1]. Conventional therapies for lymphoma include systemic chemotherapy, radiotherapy, surgery, and high-dose chemotherapy, followed by autologous stem cell transplantation (HDC/ASCT). With a better understanding of lymphoma and advances in genetic analysis, the advent of monoclonal antibodies such as rituximab or immune checkpoint inhibitors has significantly improved the outcomes of malignant lymphoma. However, the prognosis of most patients with relapsed or refractory (R/R) B-cell NHL still remains poor [2, 3]. In addition, a number of patients relapse or are refractory to the chemotherapy and/or radiotherapy, though most patients with classical HL are cured by initial therapy [4, 5].

Chimeric antigen receptor (CAR) T-cell therapy for hematologic malignancies is a breakthrough advance in the last few years. CARs are recombinant receptors for specific antigens, which include the antigen recognition and T-cell signaling domains [6–8]. When encountered with tumors, they can rapidly generate specific tumor-targeted T cells, which may exert both immediate and long-term effects [7, 9, 10]. In the USA, tisagenlecleucel and axicabtagene ciloleucel are approved for adult patients with R/R B-cell lymphomas [11–13]. In China, the dramatic development of these gene-engineered T-cell therapies also significantly improved the survival and quality of life in patients with R/R lymphoma, especially R/R NHL. In this review, we summarize the published clinical results of CAR T-cell alone or in combination with other therapies that have been conducted in mainland China, not only in R/R NHL but also in R/R HL, to bring insights into therapeutic strategies for patients with malignant lymphoma.

## Non-Hodgkin B-cell lymphoma

### CD19 CAR T-cell therapy

CD19 is expressed by essentially all B-lineage cells and regulates the intracellular signal transduction [14]. Anti-CD19 CAR T-cell therapies have shown high responses in R/R B-cell lymphoma [15, 16]. In a clinical trial investigating the efficacy and safety of CD19 CAR T-cell therapy, 22 patients with B-cell lymphoma were enrolled, including 12 patients with R/R B-cell lymphoma and 10 patients who were minimal residual disease (MRD) positive. Among them, ten patients were diagnosed with diffuse large B-cell lymphoma (DLBCL), four patients with small lymphocytic lymphoma (SLL), two patients with mantle cell lymphoma (MCL), three patients with lymphoblastic lymphoma (LBL), two patients with follicular lymphoma (FL) and one patient with mucosa-associated lymphoid tissue (MALT) lymphoma. In this clinical trial, the inclusion of MRD-positive patients was defined as that the efficacy could not reach complete remission (CR) but achieve partial remission (PR) after conventional therapies. All patients were infused with a median of 7.2 (range: 2.0 ~ 12.0) × 10^6^/kg CAR T-cells for a total of two or three times, in whom six patients received nivolumab (200 mg) once. The objective response rate (ORR) was 77.3%, including CR of 45.5% and PR of 31.8%. For 12 patients with R/R B-cell lymphoma, 2 patients achieved CR and 7 patients achieved PR. For the 5 patients who had no response to the infusion, high expression of PD-1 was exhibited. Three of those patients were treated with nivolumab and two patients achieved remission, with a patient receiving hematopoietic stem cell transplantation subsequently and the other patient sustaining remission for 12 months. For the ten MRD positive patients, eight converted to a negative status and have not relapsed for a median duration of 8 (range: 3–18) months. Among the ten patients, three received nivolumab and all achieved CR. CD19 CAR T-cells were detected to proliferate in the peripheral blood of all patients and the median time to reach the peak was 4.5 (range: 1–12) days after infusion in patients with R/R B-cell lymphoma compared with 12 (range: 5–19) days in patients who were MRD positive. 14 patients experienced cytokine release storm (CRS), including 9 patients with grade 1, 4 patients with grade 2, and 1 patient with grade 3 who had recovered after receiving an anti-IL-6 antibody and glucocorticoid [17] (Table 1**)**.

**Table 1:** Clinical trials of CAR T-cell therapy in relapsed or refractory lymphoma in China

Target antigen	Lymphoma subtype (patient number)	Patient number	Costimulatory molecule	Conditioning chemotherapy	Median dose of CAR T cells (range)	Clinical response	CRS (*n*)	Neurotoxicity (*n*)	References
Trials with single target antigen									
CD19	DLBCL (10), SLL (4), MCL (2), MALT lymphoma (1), FL (2), LBL (3)	22	4-1BB	FC	7.2 (2.0 ~ 12.0) × 10^6^/kg	ORR 77.3%, CR 45.5%, PR 31.8%	Grade 1 (9) Grade 2 (4) Grade 3 (1)	–	Mingfeng Zhao et al. 2019
CD19 (relma-cel)	DLBCL-NOS (41), DLBCL with TFL (9), PMBCL (4), FL (2), DHL/THL (3)	59	4-1BB	FC	100 × 10^6^ or 150 × 10^6^	3-month ORR 60.3%, BOR 75.9%, CR 51.7%	Grade 3 (2) Grade 4 (1)	3	Jun Zhu et al. 2020
CD19-BBz (86)	DLBCL (16), HGBL (1), FL (7), TFL (1)	26	4-1BB	–	6 patients with 3–6 × 10^6^, 8 patients with 6–19 × 10^7^, 11 patients with 2–4 × 10^8^	7 patients with CR, 8 patients with PR	Grade 1 (7)	None	Jun Zhu et al. 2019
CD20	DLBCL (8), FL (1), MCL (1), PCMZL (1)	11	CD137	CHOP (2), CHOD (1), CHODE (1), EOCH (1), FC (1), MACH (1), None (4)	0.41–1.46 × 10^7^/kg	ORR 81.8%, CR 54.5%, PR 27.3%	None	–	Wei-dong Han et al., 2016
CD30	NSHL (13), LDHL (1), MCHL (3), C-ALCL (1)	18	CD137	FC, GMC-like chemotherapy, PC	1.56 (1.1–2.1) × 10^7^/kg	7 patients with PR, 6 patients with SD, mPFS 6 months	–	–	Wei-dong Han et al. 2017
CD30	NSHL (6), ALCL (3)	9	CD28 and 4- 1BB	FC	1.4 (0.7–3.2) × 10^7^/kg	7 patients with CR	Grade 1 (3) Grade 2 (1) Grade 3 (1) Grade 5 (1)	–	J.F.Zhou et al. 2020
Trials with dual target antigens									
CD19/22	BL (2), DLBCL (23), HGBL (7), FL (3), PMBCL (1), others (2)	38	CD28 and 4–1 BB	FC	CD19 CAR T- cells, 5.1 ± 2.1 × 10^6^/kg and CD22 CAR T cells, 5.3 ± 2.4 × 10^6^/kg	ORR 72.2%, CR 50%, PR 22.2%, mPFS 9.9 months and mOS 18.0 months	Grade 1–2 (30) Grade 3–5 (8)	5	Jianfeng Zhou et al. 2020
CD22/19	DLBCL (12), FL (1), MCL (1)	14	CD28 and 4–1 BB	FC	CD22 CAR T cells, 5.6 (2.9–11.0) × 10^6^/kg and CD19 CAR T cells, 4 (2.1–8.0) × 10^6^/kg	ORR 76.9%, CR 53.8%, mPFS 6.2 months and mOS not reached	Grade 1–2 (12) Grade 3 (2)	–	Xiaoxi Zhou et al. 2020
CD19/22	DLBCL (13), BL (1), LBL (2)	16	4-1BB	FC	1–10 × 10^6^/kg CD19/22 dual-targeted CAR T- cells	ORR 87.5%, CR 62.5%, 2-year OS 77.3%,2-year PFS 40.2%	Grade 1 (4) Grade 2 (11) Grade 4 (1)	None	He Huang et al. 2021
CD19/20 (TanCAR7)	DLBCL (16), TFL (3), FL (6), others (3)	28	4-1BB	FC with or without doxorubicin liposome	0.5–8 × 10^6^/kg TanCAR7 T cells	ORR 79%, CR 71%,12-months PFS 64%	Grade 1 (8) Grade 2 (2) Grade 3 (4)	No grade 3 or higher CRES	Weidong Han et al., 2020
Trials in combination with Nivolumab									
CD19 and Nivolumab	DLBCL (10), BL (1)	11	4-1BB (CD137)	FC	8 (5–11) × 10^6^/kg, Nivolumab 3 mg/kg once	ORR 81.18%, CR 45.45%	Grade 1 (3) Grade 2 (6)	1	Mingfeng Zhao et al. 2019

*BL* Burkitt lymphoma, *C-ALCL* cutaneous ALCL, *DLBCL* diffuse large B-cell lymphoma, *DLBCL-NOS* nonspecific diffuse large B-cell lymphoma, *TFL* transformed follicular lymphoma, *PMBCL* primary mediastinal large B-cell lymphoma, *DHL/THL* double-/triple-hit lymphoma, *FC* fludarabine and cyclophosphamide, *FL* follicular lymphoma, *HGBL* high-grade B-cell lymphoma, *LBL* lymphoblastic lymphoma, *LDHL* lymphocyte-depleted Hodgkin lymphoma, *MCHL* mixed cellularity Hodgkin lymphoma, *MALT* mucosa-associated lymphoid tissue, *MCL* mantle cell lymphoma, *NSHL* nodular sclerosis Hodgkin lymphoma, *PCMZL* primary cutaneous marginal zone lymphoma, *SLL* small lymphocytic lymphoma

Relmacabtagene autoleucel (relma-cel) is a second-generation anti-CD19 CAR T-cell product with a 4-1BB co-stimulatory domain manufactured in China, which expresses the same CAR as lisocabtagene maraleucel from the USA. In a phase 1 dose-escalating trial, 23 patients were enrolled and received a single infusion of relma-cel in escalating dose levels (25 × 10^6^, 50 × 10^6^, 100 × 10^6^, and 150 × 10^6^ CAR T-cells). Based on the evaluable efficacy of 20 patients, the best ORR was 85.0% and best CRR was 75.0%. The 2-year PFS and OS rates were 60.0% and 70.0%. The most common adverse events were neutropenia (95.5%), leukopenia (95.5%), hypogammaglobulinemia (90.9%), lymphopenia (59.1%) and pyrexia (59.1%). Two patients had grade 2 CRS, while one patient experienced grade 3 CRS resolved by tocilizumab. No patients had grade ≥ 2 neurotoxicity events [18]. In the first prospective, single-arm, multicenter study, 68 patients with R/R large B-cell lymphoma (LBCL) were enrolled and 59 patients were treated with either 100 × 10^6^ (*n* = 27) or 100 × 10^6^ (*n* = 32) as a single infusion after lymphodepleting chemotherapy. Among the 58 evaluable patients, the primary end point of 3-month ORR was 60.3%. With a median follow-up of 8.9 months, median PFS and DOR were 7.0 months and 8.0 months. 28 patients (47.5%) had CRS of any grade, and grade 3 and grade 4 CRS occurred in 2 patients (3.4%) and 1 patient (1.7%). 12 patients experienced neurologic events, with only 3 patients having severe grade events [19].

### CD19 CAR T-cell therapy + nivolumab

Nivolumab, an IgG4 completely humanized anti-PD-1 monoclonal antibody, exerts a powerful antitumor ability through its programmed death ligand 1 (PD-L1). Preclinical studies have demonstrated that blockade of programmed cell death-1 (PD-1) pathway can significantly enhance the efficacy of CAR T-cell therapy against established solid cancers [20]. In a single-center study, ten patients with DLBCL and one patient with Burkitt’s lymphoma were recruited. Among them, five patients had primary refractory disease and six patients had relapsed from prior therapies. All patients received a median of 8 × 10^6^/kg (range: 5–11 × 10^6^/kg) CD19 CAR T-cell infusion in total and subsequently infused with nivolumab (3 mg/kg). The ORR was 81.18% (9/11), CR 45.45% (5/11), and the median progression-free survival (PFS) was 6 (range: 1–14) months at the median follow-up time of 6 (range: 1∼15) months. The infusions were safe and feasible. Nine patients experienced low-grade CRS, including three patients with grade 1 and six of grade 2. Only one patient presented tremors, which was resolved with symptomatic treatment. Grade 3 or 4 adverse events (AEs) were reported in 45.5% of all patients, and nearly all had cytopenia. The toxicities were manageable and reversible, and no serious immune-mediated AEs or deaths were attributable to treatment-related toxicity [21].

### A new anti-CD19 CAR (CD19-BBz (86)) T-cell therapy

The major challenges of anti-CD19 CAR T-cell therapies are severe CRS and neurotoxicity. To tackle this problem, Zhu and colleagues generated a new anti-CD19 CAR molecule (CD19-BBz (86)) derived from the CD19-BBz prototype containing both co-stimulatory 4-1BB and CD3ζ domains. In this phase I trial of CD19-BBz (86) CAR T-cell therapy, 26 patients with refractory B-cell lymphoma were enrolled and 25 patients received the infusion, in which 1 failed the trial because of an inability to manufacture a sufficient amount of CAR T cells. Six patients were infused with the lowest dose of 3–6 × 10^6^ CAR T cells and three patients had a treatment response. Eight patients received a medium dose of 6–19 × 10^7^ CAR T-cells and four patients achieved PR. 11 patients received the highest dose of 2–4 × 10^8^ CAR T- cells and 6 patients achieved CR (54.5%) and 2 achieved PR (18%). Notably, seven patients experienced CRS grade 1 and no neurological toxicities occurred. Additionally, no patient developed a dose-limiting toxicity (DLT), and the maximum tolerated dose (MTD) was not reached [22].

### Sequential infusion of CAR19/22 T-cell cocktail therapy

Relapse due to CD19 antigen loss has been a major challenge for long-term remission after anti-CD19 CAR T-cell therapy. It has been reported that the anti-CD22 CAR T-cell therapy has potency against pre-B-cell acute lymphoblastic leukemia (B-ALL), including leukemia types resistant to anti-CD19 immunotherapy [23]. In an open-label, single-center, single-arm pilot study, 89 patients who had R/R B-cell malignancies were enrolled, including 51 patients with acute lymphoblastic leukemia and 38 patients with NHL. Patients were given fludarabine (25 mg/m^2^) and cyclophosphamide (300 mg/m^2^) for 3 days (days – 4 to – 2) for lymphodepletion chemotherapy. Sequential infusion of CAR19/22 T cells were done separately on consecutive days from day 0. For 38 patients with B-cell non-Hodgkin lymphoma (B-NHL), the amount of CAR19 and CAR22 T cells was 5.1 ± 2.1 × 10^6^/kg and 5.3 ± 2.4 × 10^6^/kg, respectively. All patients with B-NHL exerted aggressive clinical courses, including 23 patients (60.5%) who relapsed more than three times or had primary refractory diseases. The ORR was 72.2%, CR 50%, and PR 22.2% at a minimum follow-up of 3 months. The median PFS was 9.9 months, and the median overall survival (OS) was 18.0 months in the B-NHL cohort. The 12-month PFS rate was 50.0% and the 12-month OS rate was 55.3%. In addition, patients at first relapse had a longer survival than those with primary refractory disease or numerous relapses. Importantly, patients who had an overall response at month 3 achieved a significantly extended PFS (*p* < 0.0001) and OS (*p* < 0.0001) compared to others [24].

Another clinical trial of sequential CD22/CD19 CAR T-cell therapy aimed to evaluate the efficacy and safety in 14 patients with R/R aggressive B-cell lymphoma involving the gastrointestinal (GI) tract. All patients had advanced diseases and received a median of 4.5 lines of prior therapies. Then they were administered intravenously with a median total dose of 5.6 × 10^6^/kg for CD22 CAR T cells, followed by 4 × 10^6^/kg of CD19 CAR T-cells. Among 13 evaluable patients, the ORR was 76.9% (10/13), CR 53.8% (7/13), and the median PFS was 6.2 months. The most common GI AEs were diarrhea (4/14, 28.6%), vomiting (3/14, 21.4%), hemorrhage (2/14, 14.3%), and nausea (2/14, 14.3%). No GI perforation occurred in any of the patients [25].

### CD19/CD22 dual-targeted CAR T-cell therapy

To conquer antigen escape-mediated relapse, bispecific CAR T cells simultaneously recognizing CD19- and CD22-expressing targets were evaluated in 16 patients. Among them, the ORR was 87.5% and CR was 62.5%. The 2-year PFS and OS were 40.2% and 77.3%, respectively. The number of prior chemotherapy lines (*n* = 2) and the response of CR were independent prognostic factors associated with favorable PFS. Only one patient experienced severe grade 4 CRS and no patients had neurotoxicity [26].

### CD20 CAR T-cell therapy

CD20 is a mature B-cell-specific molecule that functions as a membrane-embedded Ca^2+^ channel^**14**^. Therefore, CD20 antigen has been a well-established immunotherapy target for NHL derived from B cells. Wei-dong Han et al. initiated a trial to evaluate the efficacy and safety of a CD20-specific CAR T-cell therapy in patients with refractory and advanced DLBCL. Seven patients with refractory advanced CD20 + DLBCL were enrolled. Among the six evaluable patients, five patients had tumor regression. Notably, delayed toxicities were significantly associated with tumor burden and tumor-harboring sites, including CRS, sudden tumor lysis symptoms, alimentary tract hemorrhage, and aggressive intrapulmonary inflammation [27]. Thus, a phase IIa clinical trial was performed with 11 patients with R/R CD20+ B-cell lymphoma. Seven patients received systemic chemotherapy including cyclophosphamide and were infused with the CD20 CAR T-cell therapy at a dose between 0.41 × 10^7^/kg and 1.46 × 10^7^/kg. The ORR was 81.8%, 54.5% of CR, and 27.3% of PR. At a median follow-up of 8 months (range: 5–27 months), the median PFS was 6 months, and one patient had a 27-month continuous CR. There is a significant inverse correlation between the levels of the CAR gene and disease recurrence or progression. The process of CD20 CAR T-cell infusion was generally well tolerated and safe [28]. In a 5-year follow-up of these clinical trials, 12 patients with remission without autologous or allogeneic stem cell transplantation (SCT) were followed up for a median of 20 months (range: 4–60) after their first infusion of CD20 CAR T-cell therapy. The estimated median PFS was 10 months (range: 2–57) and the estimated 2-year rate of PFS was 41.7% (5/12). Regarding the delayed adverse events related to CD20 CAR T-cell therapy, no grade 4 toxicities were observed [29].

### CD19/CD20 CAR T-cell therapy

Although anti-CD19 achieved remarkable efficacy in B-cell lymphoma [11–13], a large proportion of patients experienced progressive disease partially as a result of tumor antigen loss [30]. To overcome the antigen escape by tumor cells, Weidong Han and his team designed a series of TanCARs targeting CD19 and CD20 and demonstrated that TanCAR7 achieved potent antitumor response. Further, they conducted a single-arm phase 1/2a trial involving 33 patients with R/R NHL to evaluate the safety and tolerability of TanCAR7 T cells. Among them, 28 patients received an infusion and 14 patients experienced CRS including 36% with grade 1 or 2 and 14% with grade 3. No CAR T-cell-related encephalopathy syndrome (CRES) of grade 3 or higher occurred in this study [31]. Additionally, TanCAR7 T cells were evaluated in another single-arm phase I/II trial with enrollment of 87 patients with R/R NHL, including 58 patients with aggressive DLBCL and 24 patients with high tumor burden. They received a TanCAR7 T-cell infusion at doses of 0.5–8 × 10^6^/kg. The best ORR was 78% and CR of 70%. Among patients with an objective response, 66 (97%) patients achieved the best overall response at 3 months. With a median follow-up of 27.7 months, the median PFS was 27.6 months and duration of response was not reached [32].

### Radiotherapy bridging CAR T-cell therapy

Radiotherapy has been demonstrated to be an important treatment for R/R DLBCL, especially in patients with bulky disease [33]. Ten patients with R/R DLBCL of high tumor burden were recruited in a phase II clinical trial. Six patients received local intensity-modulated radiotherapy with a total dose of 40 Gy/20 fractions in 4 weeks, while four patients were treated with systemic therapy before CAR T-cell infusion. All patients were infused with dual-target CAR T-cells based on the expression of antigens when each patient relapsed, including CD19, CD20, and CD22. The results showed that compared with patients under chemotherapy, patients in the radiotherapy group achieved a higher ORR (25% vs. 100%, *P* = 0.033) and less severe CRS (100% vs. 0%, *P* = 0.0048) and neurotoxicity (75% vs. 0%, *P* = 0.033). The study suggested that radiotherapy may be an optimal bridging regimen in patients with R/R DLBCL prior to CAR T-cell therapy [34].

## Hodgkin lymphoma and anaplastic large cell lymphoma

### CD30 CAR T-cell therapy

Hodgkin lymphoma is a unique B-cell malignant tumor characterized with scant specific malignant cells of Hodgkin cells and Reed-Sternberg cells (HRS) surrounded by numerous immunologic cells. Though nearly 90% of patients with HL will have a long-term survival [35], those with primary refractory disease or who relapsed less than 1 year from primary therapy have a worse prognosis [36].

CD30 is a member of the superfamily of tumor necrosis factor (TNF) receptor, which is consistently expressed on HRS cells and anaplastic large cell lymphoma (ALCL) cells. However, CD30 is restrictedly expressed on normal cells, making it an ideal therapeutic target in classical HL [37–39]. In a phase I clinical trial, 18 patients were enrolled, including 17 patients with heavily pretreated relapsed or refractory HL and 1 patient with relapsed primary cutaneous anaplastic large cell lymphoma (C-ALCL). All the patients received a mean of 1.56 × 10^7^ CART-30-cells/kg after conditioning regimen. After the CART-30 cell infusion, seven patients achieved PR and six patients had SD. The ORR was 39% and PFS was 6 months. The CART-30 cell infusion was safe and tolerable. The most common adverse effects related to treatment included nausea and vomiting (27.8%) and urticarial-like rash (11.1%). Only two patients had a grade 3 or higher toxicity: one patient with liver dysfunction, which may have been caused by the toxicity of the conditioning regimen, and the other patient with a left ventricular systolic function as a result of a previous megadose of adriamycin exposure [40].

In another study that evaluated the efficacy and safety of CD30 CAR T-cell therapy, nine patients with R/R CD30+ lymphoma, including six HL and three ALCL were enrolled from September 2016 to November 2018. After the lymphodepletion regimen of FC (fludarabine 25 mg/m^2^ and cyclophosphamide 20 mg/kg) for 3 days, all patients received a median dose of 1.4 × 10^7^/kg (range: 0.7–3.2 × 10^7^/kg) CD30 CAR T-cells. Seven patients achieved CR and the median PFS was 13 months and three patients with CR had a long-term survival over 2 years. Five patients with HL primarily refractory to anti-PD-1 antibody treatment were infused with anti-PD-1 antibody again. One relapsed patient regained CR and the other four patients sustained CR for at least another 8 months. Although most AEs were mild, there was a tendency that patients with more tumor burden might exert severe CRS [41].

## Conclusions

CAR T-cell therapy, a form of adoptive cellular immunotherapy, has already been tested to exert durable remissions and improve quality of life in a substantial proportion of patients with R/R lymphomas. To optimize the efficacy and safety of CAR T-cell therapy, there are still some obstacles that need to be solved. First, the resistance to CAR T-cell therapy still should be settled. Factors that influence durable remissions include CAR T-cell manufacturing issues, limited expansion and persistence in vivo, and antigen escape. Second, the immunosuppressive signals, such as cytotoxic T lymphocyte-associated protein 4 (CTLA-4) and PD-1, should be taken into consideration. Human genome editing could be a method to eliminate immunosuppressive signals and consequently enhance the function of T cells. Third, the toxicities from CAR T-cell therapy, primarily severe CRS and/or neurotoxicity, can be fatal and mitigate the efficacy. To conquer these problems, the most important tool is the proapoptotic protein caspase-9 fused to a domain of FKBP12 (inducible caspase-9) [42]. After the administration of small molecule rimiducid, the inducible caspase-9 dimerizes resulting in the rapid apoptosis of gene-engineered T cells.

To date, there are over 100 clinical trials on CAR T-cell therapy for lymphoma in China through the website *ClinicalTrials.gov*, including single antigen CAR T-cell therapy, multiple-antigens CAR T-cell therapy, or in combination with other drugs. Overall, it is imperative to further optimize CAR design and relieve the toxicities and utilize the combination with other therapies for the extensive use of this promising therapy.

## Data Availability

Not applicable.
